# Prediction of Myopia Among Undergraduate Students by Using Ensemble Machine Learning Techniques

**DOI:** 10.1002/hsr2.71425

**Published:** 2025-10-28

**Authors:** Abisha Qureshi, Laiba Shamim

**Affiliations:** ^1^ Liaquat University of Medical and Health Science Jamshoro Jamshoro Sindh Pakistan; ^2^ Jinnah Sindh Medical University Karachi Pakistan

Dear Editor,

I read the article titled, “Prediction of Myopia Among Undergraduate Students by Using Ensemble Machine Learning Techniques” by Sifat et al. [1], with great interest, which presents an innovative approach using ensemble machine learning models to predict myopia among undergraduate students in Dinajpur, Bangladesh. The authors' integration of stacking classifiers and SHAP analysis is particularly commendable, offering a noninvasive and scalable framework for predicting refractive errors in youth populations. I would like to sincerely appreciate the authors for their thoughtful research design, the clarity in methodology, and their contribution to the growing intersection of artificial intelligence and public health. However, I would like to respectfully offer a few constructive insights for further consideration.

One primary limitation of the study is the reliance on self‐reported myopia status as the primary outcome measure, which significantly limits the study's diagnostic accuracy. While practical for large‐scale data collection, self‐reporting introduces recall and confirmation bias, especially without widespread access to medical verification [2]. Future iterations of this model would benefit from incorporating clinically validated refractive error assessments, such as autorefractometry or cycloplegic refraction.

The model's impressive performance metrics (AUC = 0.979, accuracy = 95.42%) suggest potential overfitting. Although the authors used 10‐fold cross‐validation and hyperparameter tuning, the absence of external validation on an independent data set raises concerns about the model's generalizability [3]. A robust ML pipeline must include validation on geographically or demographically distinct cohorts to confirm reproducibility.

While the ensemble feature selection approach is methodologically sound, the study's external validity appears limited [4]. The model was developed and tested on a relatively homogenous sample of undergraduate students from a single region (Dinajpur, Bangladesh), which may not generalize well to broader or more diverse populations. Factors such as cultural differences, lifestyle patterns, or educational environments in other regions could significantly impact model performance. Expanding the data set to include more geographically and demographically varied populations would enhance the robustness and applicability of the findings.

Most importantly, the study acknowledges its sample limitations, yet fails to fully explore the implications of socioeconomic or academic diversity on myopia prevalence. Stratifying results by academic discipline, institution type, or family income in the modeling phase could yield deeper insights into contextual risk factors.

Despite these limitations, this study represents an important step toward accessible predictive healthcare using artificial intelligence. With refinements in data validation and broader demographic testing, the proposed framework could be instrumental in early myopia screening across resource‐limited settings. Several limitations warrant further attention. First, the data set was confined to undergraduate students from a single geographic location, limiting the generalizability of the model. Incorporating a more diverse, multiregional population would enhance the robustness and applicability of the findings. Second, the study relied on self‐reported and questionnaire‐based inputs, which may introduce recall bias or inaccuracies. Integrating clinical or biometric data could significantly strengthen predictive accuracy. Third, the cross‐sectional nature of the data restricts the ability to establish causality or track progression over time. A longitudinal study design would allow for dynamic prediction and validation of myopia development. Additionally, the lack of external validation limits confidence in the model's performance across unseen data. Addressing these limitations through broader sampling, longitudinal data collection, clinical integration, and external validation would not only improve the reliability of the model but also enhance its potential as a scalable screening tool in diverse and resource‐limited settings.

## Author Contributions

**Abisha Qureshi:** conceptualization, methodology, software, data curation, investigation, validation, formal analysis, supervision, funding acquisition, visualization, project administration, resources, writing – original draft, writing – review and editing. **Laiba Shamim:** investigation, writing – review and editing, validation, formal analysis, visualization, supervision.

## Data Availability

The data that support the findings of this study are available on request from the corresponding author. The data are not publicly available due to privacy or ethical restrictions.
